# Growth Performance, Waste Reduction Efficiency and Nutritional Composition of Black Soldier Fly (*Hermetia illucens*) Larvae and Prepupae Reared on Coconut Endosperm and Soybean Curd Residue with or without Supplementation

**DOI:** 10.3390/insects12080682

**Published:** 2021-07-29

**Authors:** Nichaphon Pliantiangtam, Pipatpong Chundang, Attawit Kovitvadhi

**Affiliations:** 1 Animal Health and Biomedical Science Program, Faculty of Veterinary Medicine, Kasetsart University, 50 Ngamwongwan Rd., Bangkok 10900, Thailand; nichaphon.p@ku.th; 2 Department of Physiology, Faculty of Veterinary Medicine, Kasetsart University, 50 Ngamwongwan Rd., Bangkok 10900, Thailand; pichandang@gmail.com

**Keywords:** *Hermetia illucens*, organic waste management, coconut endosperm, soybean curd residue

## Abstract

**Simple Summary:**

Black soldier fly (BSF, *Hermetia illucens*) larvae have a high potential to convert organic waste into high-value products. However, the growth performance, waste reduction efficiency, and chemical composition of BSF larvae are greatly influenced by the rearing substrate. This study focused on investigating the growth performance, waste reduction efficiency, and nutritional composition of BSF larvae reared on different ratios of coconut endosperm (C) and soybean curd residue (S), with or without supplementation, compared to standard diets (Gainesville: G and starter chicken diet: CK). The results showed that BSF larvae fed CK has the highest larval weight, followed by those fed coconut endosperm and soybean curd residue at a ratio of 20:80 (C20S80), and coconut endosperm and soybean curd residue at a ratio of 50:50 (C50S50) without supplementation. The greatest waste reduction efficiency was observed in the G, C50S50, and C20S80 groups without supplementation. The highest crude protein content in larvae was presented in the G and C20S80 groups followed by the CK and C50S50 groups. Therefore, equal proportions of C and S without supplementation is likely the best formulation for growth performance, waste reduction efficiency, and nutritional composition of BSF larvae when compared with standard diets.

**Abstract:**

Black soldier fly (BSF, *Hermetia illucens*) larvae are considered as insects with a high potential to convert organic waste into high-value products. The objective of this study was to investigate the growth performance, waste reduction efficiency, and nutritional composition of BSF reared on different ratios of coconut endosperm (C) and soybean curd residue (S), with or without supplementation, compared to standard diets (Gainesville: G and starter chicken diet: CK). Seven-day-old larvae were randomly divided into eight experimental groups (G, CK, and three different ratios of C and S with or without supplementation) with three replicates with an equal weight of larvae. The supplement contained calcium, phosphorus, amino acids, and a mineral–vitamin premix which was formulated to correlate with CK. Each replicate was terminated, measured, and evaluated when 40% of larvae had reached prepupal stage. The highest larval weight gain was presented in BSF fed CK, followed by those fed coconut endosperm and soybean curd residue at a ratio of 20:80 (C20S80), and coconut endosperm and soybean curd residue at a ratio of 50:50 (C50S50) without supplementation (numbers after C and S represent their percentage in the formulation; *p* < 0.001). Harvesting was delayed in the BSF fed C80S20 with and without supplementation (*p* < 0.001). The number of total larvae and prepupae was not significantly different between groups (*p* > 0.05). The greatest waste reduction efficiency was observed in the G, C50S50, and C20S80 groups without supplementation (*p* < 0.001). All groups with supplementation had a higher proportion of ash in both larvae and prepupae compared to non-supplemented groups (*p* < 0.001), but lower growth performance. The highest percentage of crude protein in larvae was presented in the Gainesville and C20S80 groups followed by the CK and C50S50 groups (*p* < 0.001). Equal proportions of C and S without supplementation are suggested as a rearing substrate. However, growth performance was lower than for CK; therefore, further studies could investigate cost-efficient techniques to promote this parameter.

## 1. Introduction

The world population has increased sharply in recent decades and could reach 9.7 billion in 2050 [1]. As a consequence, there is a higher demand for food. One-point-three billion tons of food waste is estimated to be generated per year following the sharp increase of consumption [2]. This large amount of organic waste is mainly sent to the landfill [3]. The gas released from this landfill contributes to the greenhouse effect and global warming [4,5]. It is clear from these problems that it is economically sound to study and conduct appropriate management of organic waste.

Several insects have the potential to decompose organic waste and convert it into biomass [6]. One of the most interesting insects to use as a professional decomposer is the black soldier fly (BSF), *Hermetia illucens* (Diptera: Stratiomyidae) [5,6,7]. BSF is considered a large member of the order Diptera at around 15–20 mm long [8]; it lives in the tropical zone [9], is not a disease vector, and is not harmful to humans or animals [10]. BSF larvae (feeding stage) can efficiently decompose several types of organic waste including poultry manure [11,12], cow manure [12,13], swine manure [12], human feces [13], pig’s liver waste [14], fish industrial waste [14], poultry industrial waste [13], restaurant waste [13,14,15], vegetable waste [13,15], fruit waste [16], vegetable and fruit waste [14,16,17], pineapple and jackfruit peel [18], wheat bran [13,19], maize straw [19], and beer and wine by-products [16]. In addition, BSF larvae can convert this organic waste into high-value products: protein and lipid sources for the animal feed industry [20], biodiesel [21], and antimicrobial peptides [22]. Therefore, BSF rearing could be a solution to managing and upcycling organic waste in an environmentally friendly and economically sustainable way. However, the growth performance, waste reduction efficiency, and chemical composition of BSF are greatly influenced by the rearing substrate [4,5,15]. Based on this knowledge, an appropriate rearing substrate could be used to achieve the highest decomposition efficiency and good-quality end products.

Coconut endosperm (C) and soybean curd residue (S) were used as a rearing substrate in a recent study by Lim et al. [21]; the chemical composition of rearing substrates (crude protein 8.18–20.2% DM (dry matter) and lipid 31.2–31.5% DM) that they reported was quite different from that in our study (crude protein 4.35–11.2% DM and lipid 4.69–5.61% DM), even though the same industrial by-products were used. C and S are easy to obtain in the local market or at industrial scale, as they are considered common by-products. Therefore, C and S were selected for use in this study. In most studies, good chemical composition of BSF was achieved by using chicken diet as a substrate compared to organic waste, but it is not economically sound in reality [15,23] Therefore, supplementation of organic waste with calcium, phosphorus, essential amino acids, and vitamin–mineral premix, to make it similar to chicken diet, could support the performance and quality of BSF. Based on this hypothesis, the objective of this study was to investigate the growth performance, nutritional composition, and waste reduction efficiency of BSF by rearing flies on substrates containing different ratios of C and S, with or without supplementation, compared to the standard diets (Gainesville and starter broiler chicken diet).

## 2. Materials and Methods

### 2.1. Insects, Rearing Substrates, and Chemical Analysis

Seven-day-old larvae were randomly collected from a colony of BSF larvae (Orgafeed Co., Ltd., Bangkok, Thailand) which were reared on starter broiler chicken diets (Table 1). All larvae were randomly assigned into eight experimental groups with three replicates per group (2.82 g or approximately 200 larvae per replicate). The larvae in each experimental group were fed different diets: 1. Gainesville diet (G; Scala et al. [5]); 2. Starter broiler chicken diets (CK); 3. Coconut endosperm and soybean curd residue at a ratio of 80:20 (C80S20); 4. Coconut endosperm and soybean curd residue at a ratio of 50:50 (C50S50); 5. Coconut endosperm and soybean curd residue at a ratio of 20:80 (C20S80); 6. Coconut endosperm and soybean curd residue at a ratio of 80:20 with supplementation (C80S20s); 7. Coconut endosperm and soybean curd residue at a ratio of 50:50 with supplementation (C50S50s); 8. Coconut endosperm and soybean curd residue at a ratio of 20:80 with supplementation (C20S80s). The G diet was accepted as the general experimental diet for insects in Diptera and was used as control diet in several research studies [4,5,8,12]. Therefore, the G diet was used as the control diet to compare between groups in this and other studies. The ingredients and chemical composition of diets and supplements were evaluated based on proximate analysis (AOAC 2006) and are shown in Table 1. The supplement was formulated correlating to the macro-minerals, vitamins, micro-minerals, and amino acids in the CK diet. The C and S were obtained as industrial by-products from Dutch Mill Co., Ltd. (Bangkok, Thailand). The C was treated by anaerobic fermentation for 4 weeks prior to usage [21].

### 2.2. Rearing, Data Collection, Chemical Analysis, and Calculation

The larvae were placed in a plastic container (15 cm × 24.5 cm × 6.5 cm) on the rearing substrates which were adjusted to obtain an equal humidity of 70% by analyzing the moisture in each substrates and adding water to reach the equal humidity based on calculation before providing into the container. Controlled temperature (28 ± 2 °C) and a dark room were used in this study. Each rearing container was checked twice daily at 09:30 and 16:30. Diet was added into the rearing container to achieve sufficient diet during the experiment. Each replicate was terminated when 40% of the larvae had developed into the prepupal stage [14,19]. The amount of substrate added, amount of substrate remaining, larval weight, prepupal weight, number of larvae, and number of prepupae were measured. Moreover, the pH of rearing substrates was measured at the beginning and end of the experiment by mixing rearing substrates with distilled water at 1:10 *w*/*v* [21]. The remaining substrate, larvae and prepupae were frozen and kept at −20 °C for further analysis. The larvae and prepupae were dried at 60 °C for 48 h and ground into a powder by passing through a 1-mm sieve to identify the DM, crude protein (CP), ash, and ether extract (EE), whereas the remaining rearing substrate was evaluated by DM (AOAC 2006). Substrate reduction (%SR), waste reduction index (WRI), and efficiency of conversion of digested food (ECD) were calculated following Meneguz et al. [16] and represented the formulation as below. Larval weight gain was calculated by dividing the increment in total larval weight between 7 and 14 days by seven. This study was carried out following the standard guidelines approved by the Institutional Animal Care and Use Committee of Kasetsart University, Bangkok, Thailand (ACKU63-VET-004).

(1) $$\%\mathrm{SR}=\frac{\mathrm{Distributed}\;\mathrm{substrate}\left(g\right)-\mathrm{Residual}\;\mathrm{substrate}(g)}{\mathrm{Distributed}\;\mathrm{substrate}\left(g\right)}\times 100$$

(2) $$\mathrm{WRI}=\frac{\left\{\frac{\mathrm{Distributed}\;\mathrm{substrate}\left(g\right)-\mathrm{Residual}\;\mathrm{substrate}(g)}{\mathrm{Distributed}\;\mathrm{substrate}\left(g\right)}\times 100\right\}}{\mathrm{Days}\;\mathrm{of}\;\mathrm{trial}(\mathrm{day})}$$

(3) $$\mathrm{ECD}=\frac{\mathrm{Larval}\;\mathrm{and}\;\mathrm{prepupae}\;\mathrm{weight}(g)}{\mathrm{Distributed}\;\mathrm{substrate}\left(g\right)-\mathrm{Residual}\;\mathrm{substrate}(g)}$$

### 2.3. Statistical Analysis

This experiment was performed under a completely randomized design. One-way analysis of variance (ANOVA) was performed to evaluate the differences in all measured, analyzed, and calculated data between experimental groups (fixed factors) by using Duncan’s multiple range test as post-hoc analysis. The normal distribution and homogeneity of variance were confirmed by the Shapiro–Wilk test and Levene’s test, respectively. Statistically significant difference was accepted at *p* < 0.05. All statistical analyses in the study were investigated by using the R statistics program: RStudio v1.4.1103 with the Rcmdr package (R Development Core Team 2008).

## 3. Results

The growth performance, chemical composition, waste reduction efficiency, and rearing substrate pH of BSF reared on mixed industrial by-products compared with G and CK are presented in Table 2. The highest larval weight at 14 days and larval growth rate were observed in the CK group followed by the C20S80 and C50S50 groups; the lowest performance was found in the C80S20s group (*p* < 0.001). The latest harvesting date was found for the larvae fed C and S at a ratio of 80:20 with and without supplementation; the harvesting period was around 10–11 days for other groups (*p* < 0.001). The C80S20s group had the lowest final total larval weight (*p* < 0.05). The lowest weight of each larva was presented in the C80S20s group (*p* < 0.001). There was no statistically significant difference in the total number of larvae and prepupae between groups (*p* = 0.08); however, the number of prepupae in the CK and C20S80 groups was higher than in other groups (*p* < 0.001). All groups fed C/S without supplementation (C80S20, C50S50 and C20S80) as well as the C80S20s and G groups had a higher %SR than the CK and C50S50s groups; the lowest was presented in C20S80s (*p* < 0.001). In addition, the C50S50, C20S80, and G groups had a higher WRI than the CK, C80S20, C80S20s, and C50S50 groups; the lowest was presented in C20S80s (*p* < 0.001). The greatest ECD was found in C20S80s followed by C50S50, C80S20, C20S80, C50S50s, CK, G, and C80S20s, respectively. There was a large variation in substrate pH between the beginning of the experiment and the harvesting date: 4.05–6.42 and 4.78–7.17, respectively. The lowest substrate pH at the beginning was presented in C80S20, followed by C50S50, C80S20s, C20S80, C50S50s, C80S20s, G and CK groups, respectively. Substrate pH at the end of the experiment was higher than at the beginning. The most basic substrate condition at the end of the experiment was observed in the G group followed by the CK group compared to the others (*p* < 0.001).

**Figure 1:**
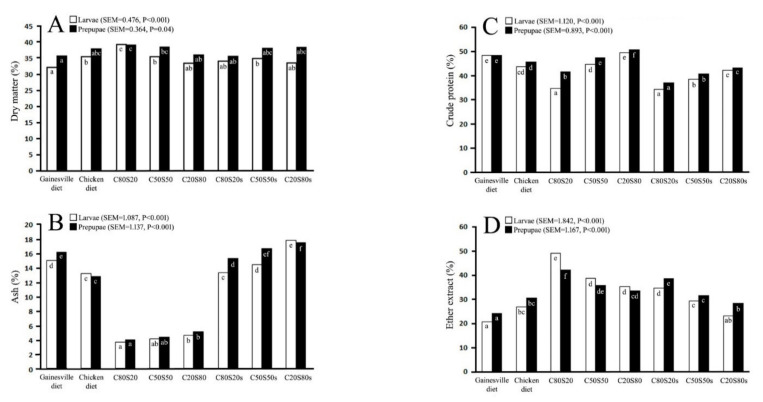
Dry matter ((**A**), %FM), ash ((**B**), %DM), crude protein ((**C**), %DM), and ether extract ((**D**), %DM) of black soldier fly larvae and/or prepupae reared on mixed industrial by-products (C: Coconut endosperm; S: soybean curd residue; number after the abbreviation represents the ratio of mixed industrial by-products) with or without supplementation (s) comparing with Gainesville and chicken diet. The statistical significant difference (*p* < 0.05) is represented by the difference of small capital letters inside bar between the experimental groups of larvae (black color) or prepupae (white color).

The chemical composition of BSF larvae and prepupae reared on mixed industrial by-products compared with G and CK is presented in Figure 1 and Supplementary Table S1. Based on the percentages of nutrients, ash content was lower in all groups fed C and S without supplementation, in both larvae and prepupae, compared to other groups (*p* < 0.001). The highest ash percentage was present in the groups fed C and S with supplementation; the highest was observed in the C20S80s group (*p* < 0.001), similar to that in CK and G groups. A high CP content in larvae and prepupae was observed in the G and C20S80 groups followed by the C50S50 and CK groups, whereas in other groups, it was lower. The lowest fat proportion in larvae and prepupae was present in the G group (*p* < 0.001). In contrast, the highest fat composition was present in the C80S20 group in both larvae and prepupae (*p* < 0.001).

## 4. Discussion

The chemical composition of rearing substrates is considered a major factor influencing the variation in growth performance, waste reduction efficiency, and nutritional composition of BSF larvae and prepupae [14,16,21,24,25]. Lim et al. [21] determined that the total final weight of larvae and growth rate were positively correlated with the amount of CP in the rearing substrate; the highest growth performance was present in larvae fed a mixture of C and S (60:40) containing 12.44% CP. The results of this study agree with those of Lim et al. [21], because the CK diet containing a high CP content (21.8%) provided a higher larval weight at 14 days and greater larval weight gain than in groups fed C and S (4.35–11.2%). Interestingly, Lim et al. [21] found that an excess CP level in the rearing substrate led to negative outcomes for these parameters, which was also represented in this study, because excess protein intake results in energy loss from metabolism involving the excretion of toxic nitrogenous waste [25]. Interestingly, the different chemical composition of the same raw materials between this study and Lim et al.’s [21] was present, but the consequences on growth performances and larval weight gain were similar. Therefore, the cause of this consequence could be influenced by several factors which could be interesting to study. However, CP content could not be the single factor which influences growth parameters. Poor BSF growth performance is found when using rearing substrates with a low caloric density, i.e., low fat and/or carbohydrate, because these nutrients serve as energy sources [23]. Generally, an appropriate ratio between CP and metabolizable energy must be formulated to obtain the highest performance in livestock animals [4]. Therefore, appropriate chemical composition of the rearing substrate could be another consideration point for BSF as it is in livestock animals. A 1:1 ratio of CP to carbohydrate for BSF cultivation has been reported to achieve the fastest development [4]. In our study, the CP:carbohydrate ratio of 0.36 in the CK diet provided the highest performance. On the one hand, C50S50 and C20S80 with CP:carbohydrate ratios of 0.23 and 0.40, respectively, presented a higher larval growth rate compared to other groups fed industrial by-products. Poor performance was found in this study when the CP:carbohydrate ratio was 0.14. However, it still cannot be concluded that only this ratio is the primary factor influencing growth rate. In our aspect, nutrient quality of amino acids and digestibility could be further studied in depth as another consideration point.

There was no significant difference in the total number of larvae and prepupae between groups in this study. We can assume that the mortality rate was not affected by experimental diets. Most studies report that the mortality rate does not change between rearing diets [21], because BSF can survive on poor nutrient diets and large environmental condition [26]. However, the duration from larvae to prepupae is prolonged when larvae are reared on diets lacking certain nutrients, mainly protein, which prolongs the cultivation period [24,25]. The feeding period is prolonged until the nutrients inside the larvae meet the requirements for development and metamorphosis at which stage they can no longer consume feed [24]. The latest harvesting date (22–23 days old) was found in the larvae fed C and S at a ratio of 80:20, which is a low protein proportion compared to that fed to other groups (which were harvested at 17 days old). In another study, the shortest rearing period (19 days old) was presented by larvae fed a mixture of C and S at a ratio of 60:40, containing the highest CP compared to other study groups [21]. In this study, the larvae being fed the CK diet for 7 days before consuming the organic waste could be the cause of an earlier harvesting date compared to the study of Lim et al. [21], in which larvae were reared on organic waste throughout the experiment [13,20]. Therefore, an appropriate starter diet could be used before rearing on organic waste to reduce rearing duration.

The pH condition of substrates did not influence the final weight [27]. However, Ma et al. [28] demonstrated that the initial substrate pH influences the final weight, the best growth performance being observed at pH 6. In the same way, Lim et al. [21] found the highest total weight and growth rate when rearing BSF at pH 5.82. In addition, it has been suggested that the rearing substrate pH should be higher than 6 to achieve a good productive performance [9]. In our study, the substrate with a pH of 6.42 (CK diet) resulted in the significantly heaviest larvae weight at 14 days, whereas a lower larvae weight was found in other groups fed industrial by-products with an initial substrate pH of 4.05–5.36. Therefore, the difference in initial substrates pH could be another cause of the diverse outcomes in this study.

The rearing substrate has a direct impact on the nutritional composition of BSF larvae and prepupae [15,16,25]. The chemical composition of the mixed organic diet and that of the BSF larvae and prepupae in this study demonstrate that the CP content in the rearing diet influences BSF larval and prepupal protein content. A high CP content in the diet produced a high protein content in larvae, a result similar to that of other studies [16]. Nitrogen-free extract (NFE) in the diet is positively correlated to the fat content in BSF larvae [25]. In this study, the increment of NFE in the mixed organic diet promoted a higher fat content in BSF larvae than in another study [15]. In addition, larvae reared on a diet high in soluble carbohydrates (618 g/kg) had the highest fat content (386 g/kg DM), whereas those reared on a diet low in soluble carbohydrates (7 g/kg) had the lowest fat content (218 g/kg DM), because insect larvae can convert excess carbohydrates to fat and store it in their body mass [16].

A great increase of ash composition (around three times) was observed in this study in both larvae and prepupae fed supplemented industrial by-products compared to the non-supplemented group. Increasing the calcium level in the substrate correlates with a higher calcium composition in house cricket (*Acheta domestica*) and yellow mealworm [29]. The supplement mainly contained calcium and phosphorus, which could be the cause of this consequence. Therefore, increasing the mineral content in the rearing substrate promoted an increase of these minerals in BSF larvae and prepupae as it did in house cricket and yellow mealworm [29]. From current knowledge from our and other studies, CK diet promotes good growth performance for BSF [15]. Therefore, the supplement was formulated to supply calcium, phosphorus, amino acids, and vitamin–mineral premix to industrial by-product diets, reflecting the CK diet which can provide benefits. However, the nutrient profiles of CK and industrial by-product diets are quite different. Our results showed that the ash content in BSF larvae and prepupae fed with supplements was similar to that in those fed CK, but there was a deterioration in growth performance compared to CK. In addition, major nutrients such as CP and carbohydrate could be more important factors influencing the growth performance than the supplement. Therefore, increasing the mineral content in rearing substrates which do not contain as appropriate a nutritive value as CK is not indicated for BSF, because a deterioration in growth performance can occur as in this study.

## 5. Conclusions

In this study, our results for growth performance, waste reduction efficiency, and the nutritional composition of BSF larvae reared on C50S50 were very similar to those for BSF larvae reared on standard diets (G and CK). However, the growth performance remained lower than in BSF fed CK. Supplementation is not necessary to improve growth performance, waste reduction efficiency, and nutritional composition when feeding larvae industrial by-product diets. Further studies could investigate the clear nutrient requirements of BSF, correlating them with their performance and quality. A technique for improving the growth performance of BSF, to reach a level similar to or better than that of larvae fed CK, by using industrial by-products as rearing substrates, could also be studied.

## Figures and Tables

**Table 1:** Ingredients and chemical composition of experimental diets.

Index	Experimental Groups							
	Gainesville diet ^1^	Chicken diet ^2^	Coconut endosperm/Soybean curd residue					
			80/20	50/50	20/80	80/20	50/50	20/80
			Supplementation ^3^					
			No	No	No	Yes	Yes	Yes
Ingredients (%as fed)								
Coconut endosperm	-	-	80	50	20	77.5	47.5	17.5
Soybean curd residue	-	-	20	50	80	17.5	47.5	77.5
Supplements ^3^	-	-	-	-	-	5	5	5
**Analyzed chemical composition (%Dry matter)**								
Dry matter (%Fresh matter)	90.5	88.2	48.4	40.7	32.4	48.8	40.8	31.9
Ash	12.3	6.40	0.70	1.15	1.85	0.68	1.13	1.90
Crude protein	17.1	21.8	4.46	6.90	11.0	4.35	6.91	11.2
Ether extract	1.70	8.00	5.60	5.27	4.73	5.61	5.28	4.69
Crude fiber	10.3	3.40	8.52	7.55	5.94	8.57	7.52	5.82
Nitrogen free extract (NFE)	52.6	60.4	31.1	29.7	27.5	31.1	29.9	27.3
Crude protein/NFE	0.33	0.36	0.14	0.23	0.40	0.14	0.23	0.40

^1^ Gainesville diet contains wheat bran, alfalfa meal, and corn meal at 50%, 30%, and 20%, respectively. ^2^ Chicken diet (Starter) contains corn meal, soybean meal, palm oil, monodicalcium phosphate, limestone, salt, vitamin–mineral premix (Feed specialties Co., Ltd.; Pathumthani, Thailand), DL-methionine, sodium bicarbonate, and choline chloride at 50.8%, 39.3%, 6.06%, 1.38%, 1.38%, 0.24%, 0.36%, 0.25%, 0.20%, and 0.07%, respectively. ^3^ Five grams of supplement contains monodicalcium phosphate, lime stone, DL-methionine, L-lysine, L-threonine, lard, and vitamin–mineral premix (Feed specialties Co., Ltd.; Pathumthani, Thailand) at 2.2 g, 1 g, 0.3 g, 0.14 g, 0.06 g, 1 g, and 0.3 g, respectively. Vitamin–mineral premix (Feed specialties Co., Ltd.; Pathumthani, Thailand) were supplied per kilogram of diets at 2,500,000 IU of vitamin A; 1,000,000 IU of vitamin D3; 7000 IU of vitamin E; 700 mg of vitamin K; 400 mg of vitamin B1; 800 mg of vitamin B2; 400 mg of vitamin B6; 4 mg of vitamin B12; 30 mg of biotin; 3111 mg of Ca pantothenate acid; 100 mg of folic acid; 15,000 mg of vitamin C; 5600 mg of vitamin B3, 10,500 mg of Zn, 10,920 mg of Fe; 9960 mg of Mn; 3850 mg of Cu; 137 mg of I; 70 mg of Se.

**Table 2:** Growth performances, waste reduction efficiency, and rearing substrate pH of black soldier fly reared on mixed industrial by-products comparing with Gainesville and chicken diet.

Parameters ^1^	Experimental Groups								SEM	*p*-Value
	Gainesville Diet	Chicken Diet	Coconut Endosperm/Soybean Curd Residue							
			80/20	50/50	20/80	80/20	50/50	20/80		
			Supplementation ^2^							
			No	No	No	Yes	Yes	Yes		
**Growth performances**										
Larval weight at 7 days (g) ^3^	2.82	2.82	2.82	2.82	2.82	2.82	2.83	2.82	0.002	0.87
Larval weight at 14 days (g) ^3^	22.7 ^c^	43.4 ^e^	17.4 ^b^	27.7 ^d^	28.2 ^d^	12.0 ^a^	17.9 ^b^	22.4 ^c^	1.896	<0.001
Larval weight gain (g/day) ^3,4^	2.85 ^c^	5.80 ^e^	2.08 ^b^	3.56 ^d^	3.62 ^d^	1.31 ^a^	2.15 ^b^	2.79 ^c^	0.271	<0.001
Duration from start to harvesting (days)	10 ^a^	10 ^a^	15 ^c^	10 ^a^	10 ^a^	16 ^c^	11 ^b^	11 ^b^	0.466	<0.001
At the end of each group ^3^										
Final fresh larval weight (g)	25.5 ^b,c^	21.0 ^a,b,c^	27.5 ^c^	28.3 ^c^	21.3 ^a,b,c^	15.0 ^a^	18.4 ^a,b^	27.7 ^c^	1.228	0.02
Final fresh larval weight (mg/larva)	159 ^c,d^	173 ^d^	138 ^b,c^	152 ^c,d^	161 ^c,d^	84.2 ^a^	122 ^b^	148 ^b,c,d^	6.110	<0.001
Final fresh prepupal weight (g)	6.93 ^a,b^	23.1 ^d^	4.95 ^a^	10.3 ^b^	15.7 ^c^	4.60 ^a^	7.38 ^a,b^	9.54 ^b^	1.262	<0.001
Final fresh prepupal weight (mg/prepupa)	100 ^a,b^	157 ^c^	110 ^a,b^	126 ^b^	124 ^a,b^	97.4 ^a^	124 ^a,b^	162 ^c^	5.305	<0.001
Number of larvae	159	122	200	187	132	173	152	187	7.285	0.05
Number of prepupae	69 ^a^	147 ^b^	45 ^a^	81 ^a^	127 ^b^	47 ^a^	59 ^a^	59 ^a^	7.649	<0.001
Number of larvae and prepupae	228	269	245	268	259	220	211	246	6.029	0.08
**Waste reduction efficiency**										
Substrate reduction (%)	49.4 ^c^	30.9 ^b^	43.9 ^c^	43.7 ^c^	49.3 ^c^	51.4 ^c^	28.9 ^b^	19.5 ^a^	2.448	<0.001
Waste reduction index (g/d) ^3^	4.94 ^c^	3.09 ^b^	2.92 ^b^	4.37 ^c^	4.93 ^c^	3.28 ^b^	2.63 ^b^	1.77 ^a^	0.235	<0.001
Efficiency of conversion of digested food	0.14 ^a,b^	0.17 ^a,b,c^	0.28 ^b,c^	0.31 ^c^	0.25 ^b,c^	0.07 ^a^	0.23 ^b,c^	0.56 ^d^	0.032	<0.001
pH of feed at										
Beginning of experiment	5.77 ^f^	6.42 ^g^	4.05 ^a^	4.21 ^b^	4.82 ^c^	4.79 ^c^	4.94 ^d^	5.36 ^e^	0.153	<0.001
Harvesting day	7.17 ^d^	6.67 ^c^	4.78 ^a^	4.97 ^a^	5.43 ^b^	4.99 ^a^	5.10 ^a,b^	5.47 ^b^	0.174	<0.001

^1^ The differences on superscripts in the same row represent the statistical significant difference at *p* < 0.05. ^2^ Each diet contains five grams of supplement comprising monodicalcium phosphate, lime stone, DL-methionine, L-lysine, L-threonine, lard, and vitamin–mineral premix (Feed specialties Co., Ltd.; Pathumthani, Thailand) at 2.2 g, 1 g, 0.3 g, 0.14 g, 0.06 g, 1 g, and 0.3 g, respectively. ^3^ Total larvae and/or prepupae were used to calculate these parameters in each study group. ^4^ Larval weight gain in fresh matter was calculated between 7- to 14 day-old larvae. Vitamin–mineral premix (Feed specialties Co., Ltd.; Pathumthani, Thailand) were supplied per kilogram of diets at 2,500,000 IU of vitamin A; 1,000,000 IU of vitamin D3; 7000 IU of vitamin E; 700 mg of vitamin K; 400 mg of vitamin B1; 800 mg of vitamin B2; 400 mg of vitamin B6; 4 mg of vitamin B12; 30 mg of biotin; 3111 mg of Ca pantothenate acid; 100 mg of folic acid; 15,000 mg of vitamin C; 5600 mg of vitamin B3, 10,500 mg of Zn, 10,920 mg of Fe; 9960 mg of Mn; 3850 mg of Cu; 137 mg of I; 70 mg of Se.

## Supplementary Materials

The following are available online at https://www.mdpi.com/article/10.3390/insects12080682/s1, Table S1: Chemical composition of black soldier fly larvae and prepupae reared on mixed industrial by-products comparing with Gainesville and chicken diet.
