# Supplementary material for: Growth Performance, Waste Reduction Efficiency and Nutritional Composition of Black Soldier Fly (Hermetia illucens) Larvae and Prepupae Reared on Coconut Endosperm and Soybean Curd Residue with or without Supplementation
Source: Insects. 2021 Jul 29;12(8):682. doi: 10.3390/insects12080682 (PMC8396427; doi:10.3390/insects12080682)
Supplement: Supplementary file 1 [file insects-12-00682-s001.zip › insects-1310544-supplementary.pdf]

Supplementary Table S1 Chemical composition of black soldier fly larvae and prepupae reared on mixed industrial by-products comparing with Gainesville and chicken diet.

Parameters [1]	Experimental Groups								SEM	*p*-Value
	Gainesville diet	Chicken diet	Coconut endosperm / Soybean curd residue							
			80/20	50/50	20/80	80/20	50/50	20/80		
			Supplementation [2]							
			No	No	No	Yes	Yes	Yes		
Chemical composition of larvae										
Dry matter (%FM)	32.2 ^{a}	35.5 ^{b}	39.4 ^{c}	35.5 ^{b}	33.5 ^{a,b}	34.2 ^{a,b}	34.9 ^{b}	33.5 ^{a,b}	0.476	<0.001
Ash (%DM)	14.7 ^{d}	12.9 ^{c}	3.58 ^{a}	4.03 ^{a,b}	4.51 ^{b}	13.1 ^{c}	14.1 ^{d}	17.4 ^{e}	1.087	<0.001
Crude protein (%DM)	48.3 ^{e}	43.7 ^{c,d}	34.8 ^{a}	44.7 ^{d}	49.4 ^{e}	34.4 ^{a}	38.5 ^{b}	42.1 ^{c}	1.120	<0.001
Ether extract (%DM)	20.7 ^{a}	26.9 ^{b,c}	49.1 ^{e}	38.7 ^{d}	35.1 ^{d}	34.7 ^{d}	29.3 ^{c}	23.1 ^{a,b}	1.842	<0.001
Chemical composition of prepupae										
Dry matter (%FM)	35.7 ^{a}	37.9 ^{a,b,c}	39.2 ^{c}	38.5 ^{b,c}	36.2 ^{a,b}	35.8 ^{a,b}	38.2 ^{a,b,c}	38.3 ^{a,b,c}	0.364	0.04
Ash (%DM)	15.8 ^{e}	12.5 ^{c}	3.93 ^{a}	4.28 ^{a,b}	5.00 ^{b}	15.0 ^{d}	16.3 ^{e,f}	17.1 ^{f}	1.137	<0.001
Crude protein (%DM)	48.4 ^{e}	45.6 ^{d}	41.7 ^{b}	47.3 ^{e}	50.7 ^{f}	36.9 ^{a}	40.8 ^{b}	43.2 ^{c}	0.893	<0.001
Ether extract (%DM)	24.1 ^{a}	30.6 ^{b,c}	42.2 ^{f}	35.8 ^{d,e}	33.4 ^{c,d}	38.7 ^{e}	31.6 ^{c}	28.4 ^{b}	1.167	<0.001

[1] The differences on superscripts in the same row represented the statistical significant difference at $p < 0.05$.

[2] Each diet contains five grams of supplement comprise monodicalcium phosphate, lime stone, DL-methionine, L-lysine, L-threonine, lard and vitamin-mineral premix[4] at 2.2g, 1g, 0.3g, 0.14g, 0.06g, 1g and 0.3 g, respectively.

[3] Vitamin-mineral premix (Feed specialties Co., Ltd; Pathumthani, Thailand) were supplied per kilogram of diets at 2,500,000 IU of vitamin A; 1,000,000 IU of vitamin D3; 7,000 IU of vitamin E; 700 mg of vitamin K; 400 mg of vitamin B1; 800 mg of vitamin B2; 400 mg of vitamin B6; 4 mg of vitamin B12; 30 mg of biotin; 3,111 mg of Ca pantothenate acid; 100 mg of folic acid; 15,000 mg of vitamin C; 5,600 mg of vitamin B3, 10,500 mg of Zn, 10,920 mg of Fe; 9,960 mg of Mn; 3,850 mg of Cu; 137 mg of I; 70 mg of Se.
